# Residential mobility among adult cancer survivors in the United States

**DOI:** 10.1186/s12889-020-09686-2

**Published:** 2020-10-23

**Authors:** Bian Liu, Furrina F. Lee, Francis Boscoe

**Affiliations:** 1 Department of Population Health Science and Policy, Icahn School of Medicine at Mount Sinai, One Gustave L. Levy Place, Box 1077, New York City, NY 10029 USA; 2 Bureau of Cancer Epidemiology, Division of Chronic Disease Prevention, New York State Department of Health, Albany, NY USA; 3 Pumphandle LLC, Portland, ME USA

**Keywords:** Cancer survivorship, Residential mobility, Neighborhood tenure, Relocation, Cancer disparities

## Abstract

**Background:**

While residential mobility affects people’s health, the dynamic of neighborhood tenure and its associated factors among cancer patients and survivors have not been studied in detail. This cross-sectional study aimed to identify sociodemographic factors associated with neighborhood tenure and relocation after the first cancer diagnosis among U.S. adult cancer survivors and patients.

**Methods:**

Based on a nationally representative sample of non-institutionalized civilian adults (≥18 years, *n* = 185,637) from the 2013–2018 National Health Interview Survey, we compared neighborhood tenure between adults with and without a history of cancer, and identified factors associated with their neighborhood tenure and relocation after the first cancer diagnosis, using propensity score matching, and logistic regression models with survey design incorporated.

**Results:**

Among adults with cancer (9.0%), 39.6% had a neighborhood tenure ≤10 years (vs. 61.2% among those without cancer), and 25.6% (equivalent to 5.4 million) relocated after their first cancer diagnosis. The odds of having shorter neighborhood tenure was higher among the cancer group in the propensity-matched samples (odds ratio = 1.05; 95% CI: 1.05–1.06; *n* = 17,259). Among cancer survivors, the odds of neighborhood relocation were negatively associated with increasing age, perceived neighborhood social cohesion, having high school level education, and being married; while positively associated with having family income below the poverty threshold, being uninsured, and living in non-Northeast regions.

**Conclusions:**

High residential mobility was found among a sizable proportion of adults with a history of cancer, and was associated with multiple socioeconomic factors. Incorporating and addressing modifiable risk factors associated with residential mobility among cancer patients and survivors may offer new intervention opportunities to improve cancer care delivery and reduce cancer disparities.

**Supplementary information:**

**Supplementary information** accompanies this paper at 10.1186/s12889-020-09686-2.

## Introduction

The places people reside throughout their lives play an important role in their health-related behaviors and their propensity to develop diseases [1]. While socioeconomic status (SES), demographic factors, environmental exposure conditions, and access to healthcare in the neighborhoods in which people reside are recognized as critical elements in promoting health and health equity [2, 3], the geospatial context of health and place has not been routinely incorporated in health research and healthcare delivery [4, 5]. In studies that have incorporated neighborhood level factors, the emphasis has largely been on the current place of residence, while little information is available regarding residential mobility, particularly in the context of cancer.

A cancer diagnosis may trigger relocation for a variety of reasons which may have a positive or negative impact on care delivery and coordination, quality of life, and ultimately survival. Relocation has been used as a proxy for household financial hardship, disruption of care continuity, and disruption of social networks, all of which carry negative impacts to health and psychosocial wellbeing, as well as survival of cancer patients [6–8]. Relocation has been found to be associated with an increased rate of late stage cancer diagnosis and an increased risk of 10-year cancer-specific mortality based on cancer registry data [6]. Geographic mobility has also been used as a proxy for social re-integration and the morbidity burden associated with mortality among survivors of childhood cancer [9]. In addition age, sex, and SES were found to be associated with mobility regardless of cancer type and relapse status [9]. Despite the potential influence of residential mobility on cancer care and survival, we know little about the dynamic patterns of relocation, reasons for relocation, and their subsequent effects on a variety of health outcomes among cancer patients and survivors.

To address these knowledge gaps, we used data from the National Health Interview Survey (NHIS) to understand residential mobility for a representative sample of the non-institutionalized U.S. civilian adult population. We aimed to compare neighborhood tenure between those with and without a history of cancer and identify sociodemographic factors associated with neighborhood tenure and relocation after the first cancer diagnosis. To the best of our knowledge, this is the first estimate of residential mobility among the non-institutionalized civilian adult population with and without cancer and identified sociodemographic factors influencing their residential mobility. Understanding these residential history patterns and risk factors, particularly modifiable ones, among cancer survivors is a first step towards a better assessment of the unmet needs among this vulnerable population. With continued growth of the cancer survivor population [10, 11], results from this study may provide novel insights into improving coordinated cancer care continuity for cancer survivors and reducing cancer disparities.

## Methods

### Data source and participants

We combined the most recent publicly available NHIS data from 2013 to 2018. NHIS is an ongoing cross-sectional household interview survey providing primary data for monitoring health and tracking progress toward achieving national health objectives [12]. Through a multistage probability clustering and stratification design, NHIS provides a national representative sample of the non-institutionalized civilian population in the United States. We included 185,637 adults aged 18 years and older who had both information on their neighborhood tenure and history of cancer. The neighborhood tenure information has been part of NHIS since 2013. Responses of “refused”, “unknown”, or “not ascertained” were treated as missing and were excluded (*n* = 4476) from the study population sample analytic domain.

### Outcome variables

The first main outcome was neighborhood tenure. Participants were asked “About how long have you lived in your present neighborhood?”, and the responses were categorized into 5 levels: less than 1 year, 1–3 years, 4–10 years, 11–20 years, and more than 20 years.

The second main outcome was neighborhood relocation after the first cancer diagnosis. We defined those who answered yes to the question “Ever told by a doctor you had cancer” as having a history of cancer. Among those with cancer, detailed questions were asked about their cancer types (up to 30 sites) and their corresponding age at cancer diagnosis. We subsequently derived the time since cancer diagnosis by subtracting age at the interview and age at the first cancer diagnosis. We categorized time since cancer diagnosis into 5 levels to match the neighborhood tenure variable. Cancer survivors were considered as having moved if their neighborhood tenure was less than their time elapsed since cancer diagnosis, and having stayed in the same neighborhood if either their time since cancer diagnosis and neighborhood tenure were in the same time duration category or if neighborhood tenure was greater than the time since cancer diagnosis.

### Covariates

We included the following sociodemographic variables: age at the interview, sex, race, Hispanic ethnicity, education, marital status, employment status, ratio of family income to the poverty threshold, health insurance coverage, residence region, nativity, family size, having family members aged 65 years and over, and having family members under 18 years of age.

We also included a measure of perceived neighborhood social cohesion (pNSC), which was assessed from four conceptually related questions [13]. Participants were asked how much they agree or disagree with the following statements about their neighborhoods: 1) “People in this neighborhood help each other out”; 2) “There are people I can count on in this neighborhood”; 3) “People in this neighborhood can be trusted”; 4) “This is a close-knit neighborhood”. The responses were on a 4-point scale: 1, definitely agree; 2, somewhat agree; 3, somewhat disagree; and 4, definitely disagree. We reverse coded the scale and summed the scores from the four questions, so that a higher total score indicated higher pNSC. These four questions have been used in other studies both on their own and in addition to a fifth question (“people in this neighborhood generally don’t get along with each other”, which was not asked in NHIS) to assess health behavior and sociological characteristics at individual and neighborhood levels [13, 14].

### Statistical analysis

We used survey procedures in SAS (V9.4) to take into account the complex NHIS sample design. Bivariate analyses were conducted to compare differences in neighborhood tenure and categorical covariates between participants with and without cancer using Rao-Scott Chi-Square tests in the *proc surveyfreq* procedure and the *proc surveymean* procedure for the two continuous variables, age and pNSC score. The distribution of neighborhood tenure and relocation after the first cancer diagnosis by major cancer types are presented as Supplemental Information (SI) in Table S1.

At the multivariable level, we used the *proc surveylogistic* procedure to fit a cumulative logit model to compare neighborhood tenure (a 5-level ordinal variable) separately for the cancer and non-cancer groups. In an attempt to adjust for confounders, we compared the differences in neighborhood tenure between cancer and non-cancer pairs identified from propensity score matching (PSM) algorithms on all 15 covariates [15, 16]. For completeness, we present the comparisons of the study population characteristics before and after PSM in Table S2 and the analysis without PSM in Table S3. Among those with a history of cancer, we fitted a binary logit model to identify factors associated with neighborhood relocation after the first cancer diagnosis. All models were adjusted for relevant covariates (unless otherwise specified), which were selected using forward selection steps (entry significance level = 0.05) in an unweighted model before inclusion in the final model. Unless otherwise noted, we report the adjusted odds ratio (aOR and 95% confidence interval, CI).

We also conducted a sensitivity analysis to identify factors associated with neighborhood relocation among a subset of cancer survivors by excluding those whose time since cancer diagnosis and neighborhood tenure were in the same time duration category, as there might be misclassification of their relocation status due to constraints of the available time resolution in the data. For example, in the scenario where the time since the first cancer diagnosis and neighborhood tenure were both 4–10 years, an actual 8-year time since cancer diagnosis and a 4-year neighborhood tenure would imply relocation.

## Results

### General characteristics of the study population (Table 1)

Of the 185,637 adults from the 2013–2018 NHIS (equivalent to a weighted 237,683,583 persons), 9% (equivalent to 21,354,205 persons) had a self-reported history of cancer. All the covariates differed significantly between those with and without a history of cancer. The weighted mean age of all samples was 47.2 years (median: 46.1 years), and participants with a history of cancer were older than their non-cancer counterparts (mean 64.5 vs. 45.5 years). A majority (86.7%) of cancer survivors reported having only one cancer, with major types being skin (non-melanoma 13.7%; melanoma 5.8%, and other 5.3%), breast (21.6%), prostate (10.7%), colon (5.4%), other (4.8%), cervix (4.6%), bladder (4.0%), lymphoma (3.5), thyroid (3.4%), uterus (2.6%), kidney (2.5%), ovary (2.2%), and lung (2.1%).

**Table 1:** Distributions of the study population characteristics overall and by cancer history status, NHIS 2013–2018

Variables	Overall (185,637, 100%)		With a history of cancer (19,105, 9.0%)		Without a history of cancer (166,532, 91.0%)	
	Unweighted N	Weighted %	Unweighted N	Weighted %	Unweighted N	Weighted %
**Neighborhood tenure (years)**						
< 1	25,568	13.1	1279	6.4	24,289	13.7
1–3	39,048	20.9	2405	12.5	36,643	21.7
4–10	46,057	25.3	4028	20.7	42,029	25.8
11–20	32,886	19.3	3875	21.2	29,011	19.2
> 20	42,078	21.4	7518	39.2	34,560	19.6
**Time since the 1st cancer diagnosis (years)**						
< 1			1270	6.3		
1–3			2394	12.5		
4–10			4015	20.7		
11–20			3857	21.2		
> 20			7489	39.2		
**Having changed neighborhoods after the 1st cancer diagnosis**						
No			13,754	74.4		
Yes			5087	25.6		
**Sex**						
Male	83,589	48.2	7890	43.9	75,699	48.7
Female	102,048	51.8	11,215	56.1	90,833	51.3
**Race**						
white only	144,910	78.8	16,858	89.4	128,052	77.7
black only	24,018	12.1	1391	6.3	22,627	12.7
AIAN only	2033	1.0	114	0.5	1919	1.0
Asian only	10,422	6.0	395	2.3	10,027	6.4
other	4254	2.1	347	1.5	3907	2.1
**Hispanic ethnicity**						
No	158,443	84.3	18,035	94.3	140,408	83.3
Yes	27,194	15.7	1070	5.7	26,124	16.7
**Education**						
< high school	24,605	12.5	2380	11.0	22,225	12.7
high school	46,359	25.0	4851	25.3	41,508	25.0
> high school	113,971	62.5	11,813	63.7	102,158	62.3
**Marital status**						
other	103,677	47.1	10,157	39.3	93,520	47.9
Yes	81,960	52.9	8948	60.7	73,012	52.1
**Employment status**						
Working	107,570	61.8	6164	35.8	101,406	64.3
Looking for work	7088	4.2	291	1.6	6797	4.4
Not working/ not looking for work	70,906	34.1	12,644	62.6	58,262	31.3
**Ratio of family income to the poverty threshold**						
< 1	27,283	12.5	1972	8.4	25,311	12.9
1–1.99	34,205	18.1	3336	16.0	30,869	18.3
2–3.99	49,136	28.8	5224	29.5	43,912	28.7
≥ 4	61,987	40.7	6943	46.1	55,044	40.1
**Health insurance coverage**						
No	21,367	11.7	644	3.5	20,723	12.5
Yes	163,589	88.3	18,437	96.5	145,152	87.5
**Residence region**						
Northeast	30,395	17.6	3291	17.8	27,104	17.6
Midwest	40,667	22.4	4444	23.5	36,223	22.2
South	66,267	36.6	6733	37.6	59,534	36.5
West	48,308	23.4	4637	21.1	43,671	23.6
**Nativity**						
Otherwise	31,407	18.6	1411	8.3	29,996	19.6
US born	154,230	81.4	17,694	91.7	136,536	80.4
**Family size**						
1	64,372	19.6	7936	24.8	56,436	19.1
2	59,669	33.6	8222	50.3	51,447	32.0
≥ 3	61,596	46.8	2947	24.9	58,649	48.9
**Having family members aged 65 and older**						
No	131,120	73.9	6957	40.3	124,163	77.3
Yes	54,517	26.1	12,148	59.7	42,369	22.7
**Having family members aged 18 and younger**						
No	132,183	65.3	16,995	85.4	115,188	63.3
Yes	53,454	34.7	2110	14.6	51,344	36.7
**People in neighborhood help each other**						
Definitely agree	72,301	39.4	8898	46.8	63,403	38.7
Somewhat agree	75,271	43.2	6885	38.4	68,386	43.6
Somewhat disagree	18,849	10.4	1589	8.6	17,260	10.5
Definitely disagree	13,275	7.0	1254	6.2	12,021	7.1
**There are people I can count on in this neighborhood**						
Definitely agree	89,638	49.0	11,088	58.5	78,550	48.0
Somewhat agree	58,230	33.1	5035	27.8	53,195	33.7
Somewhat disagree	17,309	9.6	1324	7.2	15,985	9.8
Definitely disagree	15,412	8.3	1268	6.4	14,144	8.5
**People in neighborhood can be trusted**						
Definitely agree	82,867	46.1	10,407	56.0	72,460	45.1
Somewhat agree	65,350	37.3	5745	31.8	59,605	37.8
Somewhat disagree	17,530	9.4	1293	6.8	16,237	9.6
Definitely disagree	13,525	7.2	1088	5.4	12,437	7.4
**This is a close-knit neighborhood**						
Definitely agree	52,150	28.3	6196	32.3	45,954	27.9
Somewhat agree	63,194	36.0	6222	34.1	56,972	36.2
Somewhat disagree	37,914	21.2	3611	19.6	34,303	21.3
Definitely disagree	26,981	14.5	2657	14.0	24,324	14.6
**Weighted measures**	**Mean**	**Median (IQR)**	**Mean**	**Median (IQR)**	**Mean**	**Median (IQR)**
**Age (years)**						
	47.2	46.1 (31.2–60.5)	64.5	65.5 (55.6–74.5)	45.5	43.9 (30.0–58.2)
**Perceived neighborhood social cohesion score**						
	12.2	12 (9.9–14.7)	12.7	12.9 (10.5–15.0)	12.1	11.9 (9.8–14.7)

Note: We applied survey procedures to take into account the NHIS sample design and to obtain the population weighted proportion (%) for categorical variables, and the population weighted mean, median, and interquartile range (IQR) for continuous variables. All covariates differ significantly by cancer status based on Rao-Scott Chi-Square tests for categorical variables with a *p*-value of < 0.0001, and survey regressions for continuous variables (*p*-value< 0.0001). AIAN = American Indian and Alaskan Native

The proportions of neighborhood tenure of < 1 year, 1–3 years, and 4–10 years among those with cancer were 6.4, 12.5, and 20.7%, respectively. Overall, 59.3% of the adults had resided in their neighborhood for 10 years or less, and the proportion was lower among those with than without cancer (39.6% vs. 61.2%). The proportion of those with cancer living in their neighborhood for more than 20 years nearly doubled that of those without cancer (39.2% vs. 19.6%). Approximately a quarter (25.6%, equivalent to 5,394,374 persons) of the adult cancer survivors relocated after their first cancer diagnosis. As shown in the supplemental Table S1, the proportion of having a neighborhood tenure < 1 year was the lowest among participants with a history of lung cancer (3.9%) while highest among those with cervical cancer. The proportion of relocation after the first cancer diagnosis was consistently the highest in cervical cancer (56.7%) and lowest in lung cancer (13.1%).

### Factors associated with shorter neighborhood tenure (Tables 2-3)

A similar set of factors affecting neighborhood tenure was found for both participants with and without a history of cancer (Table 2). Significant factors that were positively associated with a shorter neighborhood tenure regardless of cancer history status included: having below- poverty family income, no health insurance coverage, residing outside of the Northeast region, having a family size smaller than three, and having children in the family. On the other hand, factors negatively associated with a shorter neighborhood tenure that were shared by participants with and without a cancer history included: increasing age, having education attainment less than or equivalent to high school, being US-born, and having high perceived neighborhood social cohesion. Our analyses showed that employment status had an opposite impact on neighborhood tenure between those with and without a history of cancer. Not working/not looking-for-work (compared to working) was associated with increased odds of shorter neighborhood tenure among participants with a history of cancer (aOR = 1.15; 95%CI: 1.05–1.26), which was contrary to what was found among adults without a cancer history (aOR = 0.93; 95%CI: 0.90–0.96).

**Table 2:** Factors associated with shorter neighborhood tenure among those with and without a history of cancer, NHIS 2013–2018

Variables	With a history of cancer	Without a history of cancer
	Adjusted OR (95% CI)	Adjusted OR (95% CI)
**Age** (years)	0.95 (0.95–0.96)	0.95 (0.94–0.95)
**Race**: black only vs. white only	1.03 (0.89–1.19)	1.01 (0.97–1.06)
AIAN only vs. white only	0.92 (0.63–1.33)	0.83 (0.67–1.04)
Asian only vs. white only	0.89 (0.69–1.16)	1.06 (1.00–1.13)
Other vs. white only	1.18 (0.90–1.53)	0.99 (0.91–1.09)
**Hispanic ethnicity**: Yes vs. No	0.94 (0.79–1.11)	0.80 (0.76–0.85)
**Education**: < vs. > High school	0.82 (0.73–0.93)	0.76 (0.72–0.80)
= vs. > High school	0.82 (0.75–0.91)	0.79 (0.76–0.82)
**Employment status**: Looking for work vs. working	0.94 (0.71–1.24)	0.96 (0.89–1.03)
Not looking for work/not working vs. working	1.15 (1.05–1.26)	0.93 (0.90–0.96)
**Ratio of family income to the poverty threshold**: < 1 vs. ≥4	1.58 (1.38–1.81)	1.60 (1.51–1.68)
1–1.99 vs. ≥4	1.16 (1.03–1.30)	1.30 (1.25–1.35)
2–3.99 vs. ≥4	0.99 (0.90–1.09)	1.09 (1.06–1.13)
**Health insurance coverage**: Not covered vs. Covered	1.17 (0.96–1.43)	1.12 (1.08–1.17)
**Residence region**: Midwest vs. Northeast	1.44 (1.25–1.67)	1.38 (1.30–1.46)
South vs. Northeast	1.54 (1.35–1.77)	1.60 (1.51–1.70)
West vs. Northeast	1.80 (1.56–2.08)	1.62 (1.53–1.72)
**Nativity**: US-born vs. otherwise	0.69 (0.60–0.79)	0.64 (0.61–0.67)
**Family size**: 1 vs. ≥3	1.92 (1.64–2.25)	3.72 (3.53–3.92)
2 vs. ≥3	1.31 (1.13–1.52)	2.37 (2.26–2.49)
**Having family members aged 18 and younger**: Yes vs. No	1.83 (1.53–2.20)	2.07 (1.97–2.17)
**Perceived neighborhood social cohesion**	0.96 (0.95–0.97)	0.93 (0.93–0.94)

Notes: The response variable, neighborhood tenure, was a 5-level ordinal variable: less than 1 year (*n* = 1091), 1–3 years (*n* = 2201), 4–10 years (*n* = 3692), 11–20 years (*n* = 3557), and more than 20 years (*n* = 6718) among those with cancer; the corresponding sample sizes among those without cancer were 21,574, 34,090, 38,919, 26,503, and 30,964. The total crude sample size was 17,259 and 152,050 for those with and without cancer, respectively. Covariates were selected using forward selection steps (entry significance level = 0.05) in an unweighted model before included in the final model, where we applied survey procedures to take into account NHIS sample design

**Table 3:** Comparisons in the odds of shorter neighborhood tenure between models on data before and after the propensity-score-match

Before matching	Crude odds ratio (95% CI)	0.40 (0.39–0.42)
**Neighborhood Tenure**	**Unweighted frequency Total (Cancer / non-Cancer)**	*Weighted % for Cancer / non-Cancer
Less than 1 year	25,568 (1279/ 24,289)	6.4 / 13.7
1–3 years	39,048 (2405/ 36,643)	12.5 / 21.7
4–10 years	46,057 (4028/ 42,029)	20.7 / 25.8
11–20 years	32,886 (3875/ 29,011)	21.2 / 19.2
More than 20 years	42,078 (7518/ 34,560)	39.2 / 19.6
**After Matching**	**Crude odds ratio (95% CI)**	1.05 (1.047–1.06)
**Neighborhood Tenure**	**Unweighted frequency Total (Cancer / non-Cancer)**	*Weighted % for Cancer / non-Cancer
Less than 1 year	2130 (1091/ 1039)	5.9 / 5.6
1–3 years	4323 (2201/ 2122)	12.9 / 12.2
4–10 years	7350 (3692/ 3658)	21.0 / 20.9
11–20 years	7098 (3557/ 3541)	21.6 / 21.6
More than 20 years	13,617 (6718/ 6899)	38.6 / 39.8

Notes: The crude odds of shorter neighborhood tenure was based on a cumulative logit model using the *surveylogistic* procedure, where the response variable was neighborhood tenure, which was a 5-level ordinal variable (Reference = More than 20 years), and the explanatory variable was cancer history status (Yes vs. No). We conducted propensity-score-match using a one-to-one nearest-neighbor matching algorithm that pairs participants with closest probability (caliper =0.25) of having a history of cancer, which were conditioned on the following 15 covariates: age, sex, race, Hispanic ethnicity, education, marital status, employment status, income, health insurance coverage status, residence region, birth place, family size, having family member aged 65 and older, having family member aged 18 and younger, and perceived neighborhood social cohesion, from the survey logistic model. We matched 17,259 participants, which was 90.3% of all 19,105 available cancer samples. We then applied the *surveylogistic* procedure to the matched samples. The standard differences of the 15 variables prior to and post- matching ranged from −0.5 to 1.1, and from − 0.04 to − 0.08, respectively (details in Table S2). *, row percent. The proportional odds assumption was met

To reduce the bias in our estimate, we identified 17,259 matched cancer/non-cancer pairs (~ 90% of the 19,105 cancer cases, Table 3) based on their propensity scores on all 15 covariates. In this matched subset, we found that the odds of shorter tenure among cancer patients were higher than their non-cancer counterparts (unadjusted OR = 1.05; 95% CI: 1.05–1.06, Table 3), which was contrary to the trend found prior to PSM (unadjusted OR = 0.40; 95% CI: 0.39–0.42; *n* = 185,637, Table 3).

In models without PSM, the adjusted model from the full sample (*n* = 169,309) showed that participants with a history of cancer did not differ significantly in their neighborhood tenure from their counterparts without cancer (aOR = 1.01; 95% CI: 0.97–1.06; Supplement Table S3). A similar set of significant covariates that were positively associated with shorter neighborhood tenure (Table S3) were low family income, no health insurance coverage, residing in non-Northeastern regions, smaller family size, and having children in the family, while advanced age, Hispanic ethnicity, low education level, being unemployed, being born in the US, and having a high level of perceived neighborhood social cohesion were significantly inversely associated with shorter neighborhood tenure.

### Factors associated with neighborhood relocation after the first cancer diagnosis (Table 4)

The odds of neighborhood relocation decreased with both increasing age (aOR = 0.98; 95%CI: 0.97–0.98) and increasing pNSC scores (aOR = 0.97; 95%CI: 0.95–0.98). The odds of having moved were also lower among males than females (aOR = 0.76; 95%CI: 0.69–0.84), lower among those with a high school level education compared to those with above high school education (aOR = 0.84; 95%CI: 0.75–0.94), and lower among those who were married (aOR = 0.64; 95%CI: 0.58–0.71). On the other hand, the odds of relocation increased among those with family income below the poverty threshold (aOR = 1.37; 95%CI: 1.15–1.63), having no health insurance (aOR = 1.52; 95%CI: 1.20–1.93), and residing in non-Northeast regions. We found generally similar results in the sensitivity analysis excluding adults whose time since cancer diagnosis and neighborhood tenure were in the same time duration category (Table S4). For example, the odds of relocation were significantly lower with age (aOR = 0.97; 95%CI: 0.97–0.98) and pNSC scores (aOR = 0.96; 95%CI: 0.95–0.98), among males (aOR = 0.72; 95%CI: 0.65–0.80), among those with high school education (aOR = 0.80; 95%CI: 0.71–0.91), and among those who were married (aOR = 0.59; 95%CI: 0.53–0.65). Significantly increased odds of relocation were found among those who were not in the labor force (aOR = 1.14; 95%CI: 1.01–1.29), without health insurance coverage (aOR = 1.60; 95%CI: 1.21–1.80), and residing outside of the Northeast region.

**Table 4:** Factors associated with neighborhood relocation after first cancer diagnosis

Variables	Adjusted Odds Ratio (95% CI)
**Age** (years)	0.98 (0.97–0.98)
**Sex**: Male vs. Female	0.76 (0.69–0.84)
**Race**: black only vs. white only	0.91 (0.76–1.10)
AIAN only vs. white only	0.51 (0.26–1.01)
Asian only vs. white only	0.79 (0.56–1.12)
Other vs. white only	1.07 (0.76–1.49)
**Hispanic ethnicity**: Yes vs. No	0.88 (0.71–1.10)
**Education**: < vs. > High school	0.88 (0.76–1.03)
= vs. > High school	0.84 (0.75–0.94)
**Marital status**: Yes vs. otherwise	0.64 (0.58–0.71)
**Employment status**: Looking for work vs. working	0.90 (0.63–1.29)
Not looking for work/not working vs. working	1.12 (1.00–1.26)
**Ratio of family income to the poverty threshold**: < 1 vs. ≥4	1.37 (1.15–1.63)
1–1.99 vs. ≥4	1.10 (0.95–1.28)
2–3.99 vs. ≥4	1.00 (0.88–1.12)
**Health insurance coverage**: Not covered vs. Covered	1.52 (1.20–1.93)
**Residence region**: Midwest vs. Northeast	1.42 (1.20–1.67)
South vs. Northeast	1.41 (1.21–1.65)
West vs. Northeast	1.65 (1.40–1.94)
**Perceived neighborhood social cohesion**	0.97 (0.95–0.98)

**Notes:** The response variable was the status of having changed neighborhoods after cancer diagnosis (Yes/No: 4583/12495, total crude sample size *n* = 17,078). Covariates were selected using forward selection steps (entry significance level = 0.05) in an unweighted model before inclusion in the final model, where we applied survey procedures to take into account NHIS sample design. *AIAN* = American Indian and Alaskan Native

## Discussion

Little is known about residential mobility and its associated risk factors among cancer survivors and people in general despite the fact that neighborhood environment plays a critical role in people’s health and their health-related behaviors. Based on a large nationally representative sample, we presented a first estimate of residential mobility among the non-institutionalized civilian adult population with and without cancer, and identified sociodemographic factors influencing their residential mobility. Our results provide new insights into incorporating the spatiotemporal contexts of where people live into health research in general, and among vulnerable subpopulation groups such as cancer survivors.

We found that a quarter of the US non-institutionalized civilian adults had a neighborhood tenure between 4 and 10 years, and 59% had resided in their neighborhood for less than 10 years. Our findings of the general residential tenure are aligned with the estimates from the U.S. Census, which showed that the average 5-year moving rate in the U.S. population was approximately 35% and the median residence duration was approximately 5 years [17, 18]. The percentage of < 1 year neighborhood tenure was 13.1% overall, which is similar to that found in the general population based on census data, where 11.7% of the US population (35.9 million) had moved between 2012 and 2013 [19]. The proportion of moving for “health reasons” was small in the census data, and was collapsed into the “other” category, which only represented 2.3% of the sample, while the three major reasons for moving were family-related (30.3%), employment-related (19.4%), and housing-related (48.0%) [19]. In NHIS samples, a sizable proportion of cancer survivors (25.6%, equivalent to 5.4 million) had changed neighborhoods after their first cancer diagnosis.

Our results expand upon the descriptive epidemiology in the current literature by quantifying the associations between multiple risk factors and neighborhood tenure and relocation. Neighborhood tenure in both cancer and non-cancer groups in general was affected by a similar set of common socioeconomic and demographic factors, including age, education, income, health insurance, and family structure. Sensitivity analyses indicated these main findings were robust. These observed associations were consistent with the set of factors for neighborhood relocation among those with cancer. For example, we found that adults with family income below the poverty threshold, with a lower level of education and without health insurance coverage were more likely to move after their first cancer diagnosis. Among cancer survivors, detailed reasons of neighborhood tenure and relocation may differ by cancer status (e.g. cancer types, cancer treatment and survival status). We found that the proportion of relocation among participants with a history of lung cancer, which has a low survival rate (5-year survival rate ~ 20% [20]), was one quarter of that among participants with a history of cervical cancer (5-year survival rate 66% [21]).

Despite the similarities in neighborhood tenure between people with and without a history of cancer, cancer survivors may face unique challenges. This is supported by results from the propensity-score-matched analysis, where cancer survivors experienced increased odds of shorter neighborhood tenure than their non-cancer counterparts. A relocation may result from or inflict a larger health, psychosocial, and economic burden among cancer survivors than those without cancer. Our stratified analysis showed that unemployment status increased the odds of shorter neighborhood tenure among cancer survivors, while the opposite was true for non-cancer adults. Changes in employment status may be a direct result of a cancer diagnosis and its related treatment, which tend to adversely affect cancer survivors. Work ability and performance [22, 23]. Moreover, changes in employment status may also adversely contribute to financial toxicity among cancer survivors, such as reduced income and loss of employer-sponsored health insurance coverage [23, 24], all of which may in turn prompt a move. For people who were already facing financial hardship predating a cancer diagnosis, relocation may add additional strain and psychosocial stress. Qualitative investigations have found a potential spiral to acute and irreversible financial stress among patients who had to relocate for specialist treatment for hematological malignancy [25]. Other studies have demonstrated extra financial burden and physiologic stress associated with cancer diagnosis and treatment that led to delaying or forgoing treatments and medications, reduced quality of life, and ultimately mortality [26–31]. However, few studies have investigated the sequelae of residential mobility among cancer patients and survivors. We were only able to find one such study, where a recent relocation (within 3 years) prior to a second cancer diagnosis was associated with an approximately 30% increased risk of late-stage presentation and a 26% increase in 10-year cancer-specific mortality, and the negative impact was larger for patients living in the least wealthy counties [6].

It stands to reason to postulate that a shorter residential tenure and neighborhood relocation may cast a stronger negative impact on the overall well-being of cancer survivors, as residential mobility is closely related to household financial hardship, disruption of care continuity, and disruption of social networks [6–8, 10]. Alternatively, a relocation to be closer to family members and friends, closer to cancer treatment facilities, to a more affordable and/or friendly neighborhood, to be closer to a job, or a combination of these, may also help alleviate the financial and psychosocial stress among cancer survivors. Our analysis showed that those who considered their own neighborhoods to have strong social cohesion were more likely to stay in the neighborhood. This previously unreported finding may have potential implications in improving care and outcomes while reducing disparities in cancer survivorship. Indeed, longitudinal studies have shown that the overall, cardiovascular disease-specific, and cancer-specific mortality rates were positively associated with neighborhood improvement while negatively associated with neighborhood deterioration [32].

### Limitations

The study shares some of the same limitations of other studies that have used NHIS data. First, the cross-sectional nature of the survey and the lack of information regarding the reason for relocating prevented us from drawing causal inferences. Rather, the results are better served for hypothesis generating purposes. Second, our results are not generalizable to the institutionalized or non-civilian populations, such as adults in a skilled nursing facility or the military/veteran population. In addition, cancer survivors who were still alive at the time of the interview were different from patients with cancers with a less favorable prognosis. Cancer survivors with more advanced disease were more likely to live in nursing homes, long-term-care facilities, or hospice, and thus were not included in the survey. Mortality data were not available in the public NHIS data release, thus the subsequent health impact of neighborhood tenure and relocation on survival could not be explored in the current study, and warrants further investigation. Other factors such as availability and accessibility of oncological infrastructure, which influence residential mobility of cancer patients but were not available in the data, were not considered in the current analysis. Finally, all the measures were based on self-reported responses, and were subject to recall and response bias. Nonetheless, the large sample size and national representativeness of the NHIS data allowed us to provide the first estimate, to our knowledge, of the residential mobility patterns among adult cancer survivors. Given the dearth of information on this topic, our study lends evidence to future studies to examine detailed dynamics of residential mobility and their downstream impact on cancer survivorship.

## Conclusion

With continued improvement in early detection and treatment options for cancer, the proportion of people who have had a cancer diagnosis is expected to grow. Our estimates of residential mobility among adult cancer survivors are critical to inform policies and practices aimed at improving survivorship care and outcomes as well as reducing cancer disparities. Shorter neighborhood tenure and neighborhood relocation may increase financial hardship, disrupt cancer patients’ social and care support networks, and interrupt continuity of care. These negative impacts are potentially greater among those with low socioeconomic status. On the other hand, sound neighborhood built environments including high social cohesion can provide positive impacts among cancer survivors. To examine the long-term implications for health and economic well-being associated with cancer, awareness of residential mobility, an important aspect that has often been neglected, should be incorporated into medical care research and practice.

## Supplementary information

**Additional file 1: Table S1.** Distributions of neighborhood tenure and relocation by major cancer types, NHIS 2013–2018. **Table 2.** Standard differences in variables before and after the propensity score matching. **Table S3.** Associations between shorter neighborhood tenure and cancer history status. **Table S4.** Factors associated with neighborhood relocation after the first cancer diagnosis, excluding those whose cancer duration and neighborhood tenure were in the same time duration category.

## Data Availability

Data used for this study were publicly available from the NIHS website, https://www.cdc.gov/nchs/nhis/data-questionnaires-documentation.htm
