# Supplementary material for: Residential mobility among adult cancer survivors in the United States
Source: BMC Public Health. 2020 Oct 23;20:1601. doi: 10.1186/s12889-020-09686-2 (PMC7585207; doi:10.1186/s12889-020-09686-2)
Supplement: Supplementary file 1 — Additional file 1: Table S1. Distributions of neighborhood tenure and relocation by major cancer types, NHIS 2013–2018. Table 2. Standard differences in variables before and after the propensity score matching. Table S3. Associations between shorter neighborhood tenure and cancer history status. Table S4. Factors associated with neighborhood relocation after the first cancer diagnosis, excluding those whose cancer duration and neighborhood tenure were in the same time duration category. [file 12889_2020_9686_MOESM1_ESM.docx]

**Supplemental Information**

**Table S1.** Distributions of neighborhood tenure and relocation by major cancer types, NHIS 2013-2018.

| **Cancer types** | **Neighborhood tenure (years)** | **Unweighted N (weighted%)** | **Moved after diagnose of 1^st^ cancer diagnosis** | **Unweighted N (weighted%)** |
| --- | --- | --- | --- | --- |
| **Breast** | <1 | 196 (4.8) | No | 2637 (76.3) |
|  | 1-3 | 431 (11.4) | Yes | 901 (23.7) |
|  | 4-10 | 695 (19.6) |  |  |
|  | 11-20 | 749 (22) |  |  |
|  | >20 | 1501 (42.2) |  |  |
| **Bladder** | <1 | 29 (4.5) | No | 404 (81.2) |
|  | 1-3 | 60 (13.4) | Yes | 105 (18.8) |
|  | 4-10 | 77 (14.2) |  |  |
|  | 11-20 | 118 (20.5) |  |  |
|  | >20 | 227 (47.4) |  |  |
| **Cervix** | <1 | 157 (13.1) | No | 436 (43.3) |
|  | 1-3 | 228 (23.4) | Yes | 604 (56.7) |
|  | 4-10 | 292 (26.2) |  |  |
|  | 11-20 | 147 (15.1) |  |  |
|  | >20 | 218 (22.2) |  |  |
| **Colon** | <1 | 55 (5.1) | No | 819 (80.1) |
|  | 1-3 | 112 (12.3) | Yes | 224 (19.9) |
|  | 4-10 | 214 (20.5) |  |  |
|  | 11-20 | 208 (20.7) |  |  |
|  | >20 | 463 (41.4) |  |  |
| **Kidney** | <1 | 28 (6.7) | No | 296 (80.7) |
|  | 1-3 | 54 (15.2) | Yes | 77 (19.3) |
|  | 4-10 | 78 (20.2) |  |  |
|  | 11-20 | 71 (19.8) |  |  |
|  | >20 | 145 (38.2) |  |  |
| **Lung** | <1 | 26 (3.9) | No | 407 (86.9) |
|  | 1-3 | 58 (11.7) | Yes | 70 (13.1) |
|  | 4-10 | 99 (20.7) |  |  |
|  | 11-20 | 96 (21.1) |  |  |
|  | >20 | 201 (42.6) |  |  |
| **Lymphoma** | <1 | 41 (7.8) | No | 369 (71.4) |
|  | 1-3 | 55 (12.5) | Yes | 144 (28.6) |
|  | 4-10 | 110 (21.2) |  |  |
|  | 11-20 | 110 (21.5) |  |  |
|  | >20 | 202 (36.9) |  |  |
| **Prostate** | <1 | 92 (4.3) | No | 1667 (84.6) |
|  | 1-3 | 190 (8.7) | Yes | 328 (15.4) |
|  | 4-10 | 342 (16.3) |  |  |
|  | 11-20 | 383 (20.6) |  |  |
|  | >20 | 1010 (50.1) |  |  |
| **Thyroid** | <1 | 33 (6.5) | No | 273 (69.9) |
|  | 1-3 | 60 (14.9) | Yes | 127 (30.1) |
|  | 4-10 | 111 (26.6) |  |  |
|  | 11-20 | 97 (25.6) |  |  |
|  | >20 | 104 (26.4) |  |  |
| **Uterus** | <1 | 82 (8.4) | No | 554 (60.2) |
|  | 1-3 | 148 (15.3) | Yes | 391 (39.8) |
|  | 4-10 | 253 (27.5) |  |  |
|  | 11-20 | 164 (15.7) |  |  |
|  | >20 | 308 (33.1) |  |  |
| **Skin (Melanoma)** | <1 | 86 (6) | No | 926 (75.4) |
|  | 1-3 | 153 (11.5) | Yes | 320 (24.6) |
|  | 4-10 | 256 (22) |  |  |
|  | 11-20 | 278 (23.1) |  |  |
|  | >20 | 484 (37.4) |  |  |
| **Skin (non-melanoma)** | <1 | 173 (5) | No | 2511 (77.1) |
|  | 1-3 | 366 (11.1) | Yes | 824 (22.9) |
|  | 4-10 | 693 (19.4) |  |  |
|  | 11-20 | 778 (24.2) |  |  |
|  | >20 | 1356 (40.3) |  |  |
| **Skin (other)** | <1 | 81 (6.7) | No | 962 (77.6) |
|  | 1-3 | 151 (11.7) | Yes | 317 (22.4) |
|  | 4-10 | 272 (19.6) |  |  |
|  | 11-20 | 232 (17.4) |  |  |
|  | >20 | 561 (44.6) |  |  |
| **Other cancer** | <1 | 200 (9.6) | No | 1493 (69.5) |
|  | 1-3 | 339 (14.1) | Yes | 655 (30.5) |
|  | 4-10 | 536 (23.3) |  |  |
|  | 11-20 | 444 (21.6) |  |  |
|  | >20 | 738 (31.4) |  |  |

**Note:** We applied survey procedures to take into account NHIS sample design, and to obtain population weighted proportion (%) for neighborhood tenure and relocation across major cancer types. The distributions differed significantly by cancer types based on Rao-Scott Chi-Square tests for neighborhood tenure (p-value <0.0001) and neighborhood relocation (p-value <0.0001).

**Table S2.** Standard differences in variables before and after the propensity score matching.

| **Variables** | **Before matching** | **After matching** |
| --- | --- | --- |
| Age | 1.1075 | 0.0138 |
| Sex | -0.084 | 0.0121 |
| Race | 0.3224 | 0.0824 |
| Hispanic ethnicity | -0.3315 | 0.0306 |
| Education | 0.031 | 0 |
| Marital Status | 0.0602 | -0.0089 |
| Employment | 0.6522 | 0.0835 |
| Ratio of family income to poverty threshold | 0.1574 | 0.0346 |
| Health insurance coverage | -0.3423 | 0.0162 |
| Residence region | 0.0415 | 0 |
| Nativity | 0.3233 | -0.0291 |
| Family size | 0.4776 | 0.0298 |
| Having family members aged 65 years and older | 0.8311 | 0.0293 |
| Having family members aged 18 years and younger | -0.5014 | 0.0225 |
| Neighborhood cohesion | 0.1704 | -0.0414 |

**Note:** We conducted propensity-score-match using a one-to-one nearest neighbor matching algorithm that pairs participants with closest probability (caliper =0.25) of having a history of cancer, which were conditioned on 15 covariates, using method by Coa-Perraillon (https://support.sas.com/resources/papers/proceedings/proceedings/forum2007/185-2007.pdf). We matched 17,259 participants, which was 90.3% of all 19,105 available cancer samples. Comparison of the distribution of characteristics before and after matching were conducted using the method by Yan and Dalton (http://support.sas.com/resources/papers/proceedings12/335-2012.pdf).

**Table S3.** Associations between shorter neighborhood tenure and cancer history status.

| **Variables** | **Adjusted odds ratio**  **(95% CI)** |
| --- | --- |
| **Cancer history**: Yes vs. No | 1.01 (0.97 - 1.06) |
| **Age** (years) | 0.95 (0.94 - 0.95) |
| **Race**:  black only vs. white only | 1.02 (0.97- 1.07) |
| AIAN only vs. white only | 0.84 (0.67- 1.04) |
| Asian only vs. white only | 1.06 (0.99- 1.12) |
| Other vs. white only | 1.01 (0.92- 1.10) |
| **Hispanic ethnicity**: Yes vs. No | 0.81 (0.76- 0.85) |
| **Education**:  < vs. > High school | 0.76 (0.73- 0.80) |
| = vs. > High school | 0.79 (0.77- 0.82) |
| **Employment status**:  Looking for work vs. working | 0.96 (0.89- 1.03) |
| Not looking for work/not working vs. working | 0.95 (0.92- 0.98) |
| **Ratio of family income to the poverty threshold**: < 1 vs. ≥4 | 1.59 (1.51- 1.68) |
| 1-1.99 vs. ≥4 | 1.29 (1.24- 1.34) |
| 2-3.99 vs. ≥4 | 1.09 (1.05- 1.12) |
| **Health insurance coverage**: Not covered vs. Covered | 1.13 (1.08- 1.17) |
| **Residence region**:  Midwest vs. Northeast | 1.38 (1.31- 1.46) |
| South vs. Northeast | 1.59 (1.51- 1.69) |
| West vs. Northeast | 1.63 (1.54- 1.73) |
| **Nativity**: US-born vs. otherwise | 0.64 (0.62- 0.67) |
| **Family size**:  1 vs. ≥3 | 3.53 (3.35- 3.71) |
| 2 vs. ≥3 | 2.26 (2.16- 2.37) |
| **Having family members aged 18 and younger**:  Yes vs. No | 2.02 (1.93- 2.12) |
| **Perceived neighborhood social cohesion** | 0.94 (0.93 - 0.94) |

**Notes:** Response variable was an ordinal variable indicating neighborhood tenure: less than 1 year (n=22,665), 1-3 years (n=36,291), 4-10 years (n=42,611), 11-20 years (n=30,060), and more than 20 years (n=37,682). The total crude sample size n= 169,309 (status of cancer history: Yes/No: 17,259/152,050). Covariates were selected using forward selection steps (entry significance level= 0.05) in an unweighted model before included in the final model, where we applied survey procedure to take into account NHIS sample design. AIAN=American Indian and Alaskan Native. The proportional odds assumption was met.

**Table S4.** Factors associated with neighborhood relocation after the first cancer diagnosis, excluding those whose cancer duration and neighborhood tenure were in the same time duration category.

| **Variables** | **Adjusted Odds Ratio**  **(95% CI)** |
| --- | --- |
| **Age** (years) | 0.97 (0.97 - 0.98) |
| **Sex**: Male vs. Female | 0.72 (0.65 - 0.80) |
| **Race**: black only vs. white only | 0.91 (0.75 - 1.11) |
| AIAN only vs. white only | 0.55 (0.25 - 1.19) |
| Asian only vs. white only | 0.81 (0.54 - 1.20) |
| Other vs. white only | 1.09 (0.74 - 1.62) |
| **Hispanic ethnicity**: Yes vs. No | 0.90 (0.71 - 1.13) |
| **Education**: < vs. > High school | 0.86 (0.73 - 1.01) |
| = vs. > High school | 0.80 (0.71 - 0.91) |
| **Marital status**: Yes vs. otherwise | 0.59 (0.53 - 0.65) |
| **Employment status**: Looking for work vs. working | 0.80 (0.53 - 1.21) |
| Not looking for work/not working vs. working | 1.14 (1.01 - 1.29) |
| **Ratio of family income to the poverty threshold**: < 1 vs. ≥4 | 1.48 (1.22 -1.80) |
| 1-1.99 vs. ≥4 | 1.10 (0.94 - 1.28) |
| 2-3.99 vs. ≥4 | 1.03 (0.91 - 1.17) |
| **Health insurance coverage**: Not covered vs. Covered | 1.60 (1.21 - 2.11) |
| **Residence region**: Midwest vs. Northeast | 1.45 (1.21 - 1.73) |
| South vs. Northeast | 1.48 (1.25 - 1.76) |
| West vs. Northeast | 1.75 (1.46 - 2.10) |
| **Perceived neighborhood social cohesion** | 0.96 (0.95 - 0.98) |

**Notes:** Response variable was the status of having changed neighborhoods after cancer diagnosis (Yes/No: 4583/8743, total crude sample size n= 13,326). Different from the main model (Table 5), in this sensitivity analysis, cancer patients whose cancer duration and neighborhood tenure were in the same time duration category were excluded (n=3752), though results were similar to those in the main model. Covariates were selected using forward selection steps (entry significance level= 0.05) in an unweighted model before included in the final model, where we applied survey procedure to take into account NHIS sample design. AIAN=American Indian and Alaskan Native.
